# Supplementary material for: iEnhancer-ELM: improve enhancer identification by extracting position-related multiscale contextual information based on enhancer language models
Source: Bioinform Adv. 2023 Mar 25;3(1):vbad043. doi: 10.1093/bioadv/vbad043 (PMC10125906; doi:10.1093/bioadv/vbad043)
Supplement: vbad043_Supplementary_Data [file vbad043_supplementary_data.pdf]

Supplementary Files

Table S1. Performance comparison between different scale *k*-mers and widely-used features for enhancer identification on the Liu's training dataset with 5-fold cross validation.

Methods	Acc	Sn	Sp	MCC
k-mer[a]	0.7410	0.7460	0.7370	0.4840
Spectrum[b]	0.7480	0.6340	**0.8620**	0.5090
Mismatch[b]	0.7590	0.7370	0.7820	0.5200
PseDNC[b]	0.7620	0.7340	0.7890	0.5250
Word2Vec[a]	0.7030	0.6930	0.7120	0.4060
FastText[a]	0.7530	0.7610	0.7440	0.5050
BERT-Enhancer[a]	0.7620	0.7950	0.7300	0.5250
iEnhancer-ELM(3-mer)	0.7946	0.7449	0.8433	0.5914
iEnhancer-ELM(4-mer)	0.7895	0.7742	0.8063	0.5800
iEnhancer-ELM(5-mer)	**0.8013**	**0.8095**	0.7933	**0.6029**
iEnhancer-ELM(6-mer)	0.7909	0.7189	0.8558	0.5820

[a] These results are from Le et al. (2021).
[b] These results are from Cai et al. (2021).

Table S2. The performance of pre-trained enhancer language models with different scale *k*-mers on Liu's test dataset.

k-mers	Acc	Sn	Sp	MCC	AUC
3-mer	0.7525	0.7300	0.7750	0.5055	0.8352
4-mer	0.7625	0.7150	0.8100	0.5274	0.8284
5-mer	0.7475	0.6800	0.8150	0.4996	0.8213
6-mer	0.7750	0.7400	0.8100	0.5514	0.8263

Table S3. The performance of pre-trained enhancer language models with different scale k-mers on Basith's dataset.

**cell line**	**k-mers**	**Bacc**	**Sn**	**Sp**	**MCC**	**AUC**
HEK293	3-mer	**0.8304**	**0.8325**	0.8283	0.6381	**0.9122**
	4-mer	0.8193	0.7814	**0.8573**	0.6289	0.9037
	5-mer	0.8175	0.7908	0.8443	0.6210	0.9024
	6-mer	0.8144	0.7833	0.8456	0.6161	0.8981
NHEK	3-mer	**0.7193**	0.6985	**0.7296**	0.4099	0.7898
	4-mer	0.6908	**0.7928**	0.6398	0.4079	0.7890
	5-mer	0.6840	0.7909	0.6305	0.3974	0.7835
	6-mer	0.6940	0.7704	0.6559	0.4020	0.7777
K652	3-mer	0.7834	0.8120	**0.7549**	0.5392	**0.8612**
	4-mer	**0.7864**	**0.8584**	0.7143	**0.5406**	0.8599
	5-mer	0.7788	0.8416	0.7160	0.5267	0.8506
	6-mer	0.7717	0.8243	0.71908	0.5138	0.8465
GM12878	3-mer	0.8177	0.7436	0.8918	0.6421	**0.9176**
	4-mer	**0.8217**	0.7521	0.8913	**0.6486**	0.9157
	5-mer	0.8092	**0.7632**	0.8553	0.6109	0.9075
	6-mer	0.8122	0.7309	0.8935	0.6338	0.9043
HMEC	3-mer	0.7525	0.7465	0.7585	**0.4841**	0.8307
	4-mer	0.7497	0.7220	**0.7774**	0.4829	0.8317
	5-mer	0.7539	**0.7855**	0.7223	0.4815	0.8296
	6-mer	0.7489	0.7593	0.7384	0.4743	0.82342
HSMM	3-mer	**0.7273**	**0.7862**	0.6684	**0.4289**	**0.7996**
	4-mer	0.7123	0.7171	**0.7076**	0.4039	0.7915
	5-mer	0.7171	0.7572	0.6770	0.4102	0.7892
	6-mer	0.7176	0.7546	0.6806	0.4113	0.7848
NHLF	3-mer	0.7456	0.8135	0.6777	0.4632	0.8217
	4-mer	0.7516	**0.8363**	0.6669	0.4744	**0.8318**
	5-mer	0.7443	0.8287	0.6599	0.4606	0.8124
	6-mer	0.7300	0.7652	0.6948	0.4352	0.7991
HUVEC	3-mer	0.7271	0.7800	**0.6743**	0.4288	**0.8062**
	4-mer	0.7329	0.8077	0.6581	0.4392	0.8033
	5-mer	0.7288	0.7956	0.6620	0.4315	0.8017
	6-mer	0.7253	**0.8120**	0.6385	0.4249	0.7938

Table S4. The ensemble performance of pre-trained enhancer language models on Basith's dataset.

cell line	k-mers	Bacc	Sn	Sp	MCC	AUC
HEK293	3-mer	**0.8304**	**0.8325**	0.8283	0.6381	**0.9122**
	4-mer	0.8193	0.7814	**0.8573**	0.6289	0.9037
	5-mer	0.8175	0.7908	0.8443	0.6210	0.9024
	6-mer	0.8144	0.7833	0.8456	0.6161	0.8981
NHEK	3-mer	**0.7193**	0.6985	**0.7296**	0.4099	0.7898
	4-mer	0.6908	**0.7928**	0.6398	0.4079	0.7890
	5-mer	0.6840	0.7909	0.6305	0.3974	0.7835
	6-mer	0.6940	0.7704	0.6559	0.4020	0.7777
K652	3-mer	0.7834	0.8120	**0.7549**	0.5392	**0.8612**
	4-mer	**0.7864**	**0.8584**	0.7143	**0.5406**	0.8599
	5-mer	0.7788	0.8416	0.7160	0.5267	0.8506
	6-mer	0.7717	0.8243	0.71908	0.5138	0.8465
GM12878	3-mer	0.8177	0.7436	0.8918	0.6421	**0.9176**
	4-mer	**0.8217**	0.7521	0.8913	**0.6486**	0.9157
	5-mer	0.8092	**0.7632**	0.8553	0.6109	0.9075
	6-mer	0.8122	0.7309	0.8935	0.6338	0.9043
HMEC	3-mer	0.7525	0.7465	0.7585	**0.4841**	0.8307
	4-mer	0.7497	0.7220	**0.7774**	0.4829	0.8317
	5-mer	0.7539	**0.7855**	0.7223	0.4815	0.8296
	6-mer	0.7489	0.7593	0.7384	0.4743	0.82342
HSMM	3-mer	**0.7273**	**0.7862**	0.6684	**0.4289**	**0.7996**
	4-mer	0.7123	0.7171	**0.7076**	0.4039	0.7915
	5-mer	0.7171	0.7572	0.6770	0.4102	0.7892
	6-mer	0.7176	0.7546	0.6806	0.4113	0.7848
NHLF	3-mer	0.7456	0.8135	0.6777	0.4632	0.8217
	4-mer	0.7516	**0.8363**	0.6669	0.4744	**0.8318**
	5-mer	0.7443	0.8287	0.6599	0.4606	0.8124
	6-mer	0.7300	0.7652	0.6948	0.4352	0.7991
HUVEC	3-mer	0.7271	0.7800	**0.6743**	0.4288	**0.8062**
	4-mer	0.7329	0.8077	0.6581	0.4392	0.8033
	5-mer	0.7288	0.7956	0.6620	0.4315	0.8017
	6-mer	0.7253	**0.8120**	0.6385	0.4249	0.7938

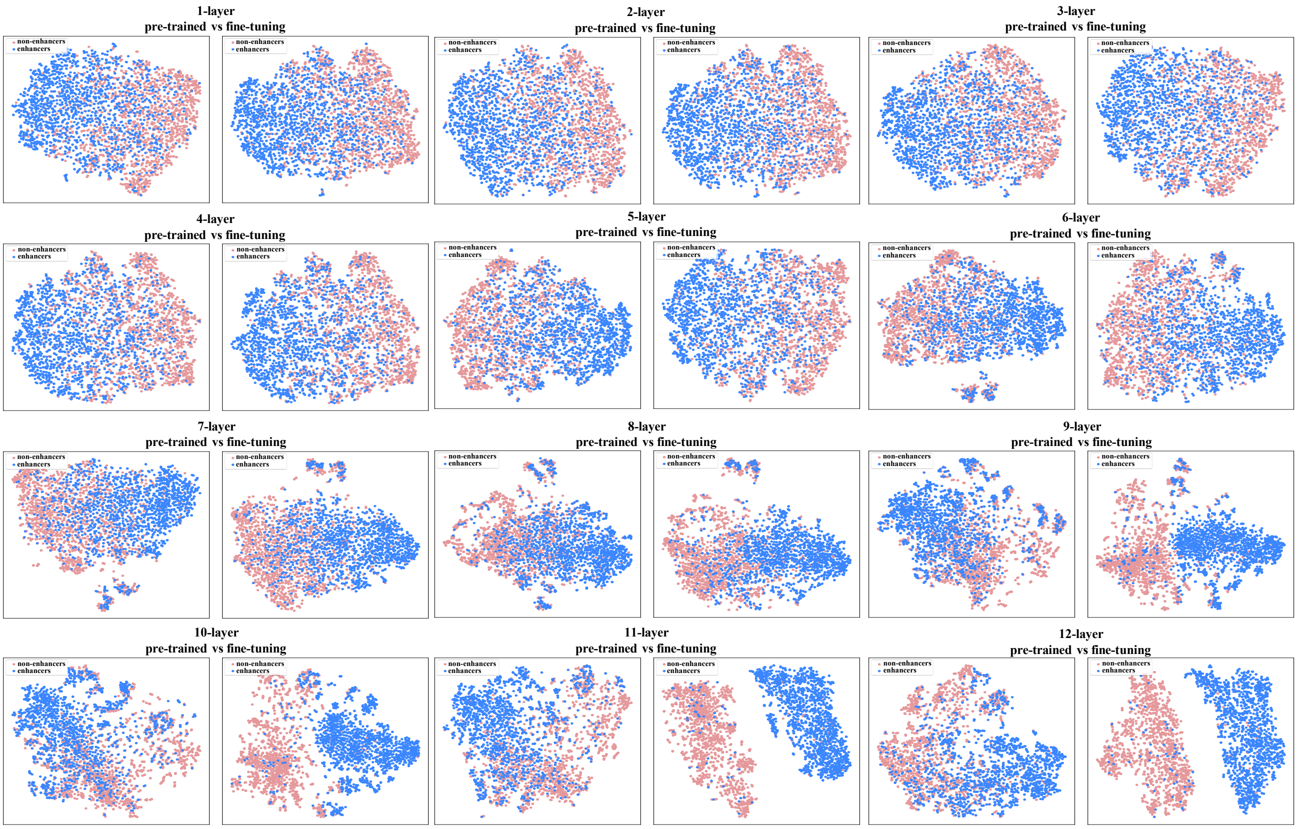

Fig. S1. Distribution comparison of enhancers and non-enhancers projected by all encoder layers of pre-trained and fine-tuned models based on 3-mer on the Liu's training dataset. In each subfigure, the left panel is the distribution projected by all encoder layers of pre-trained models, and the right panel is the distribution projected by all encoder layers of fine-tuned models.

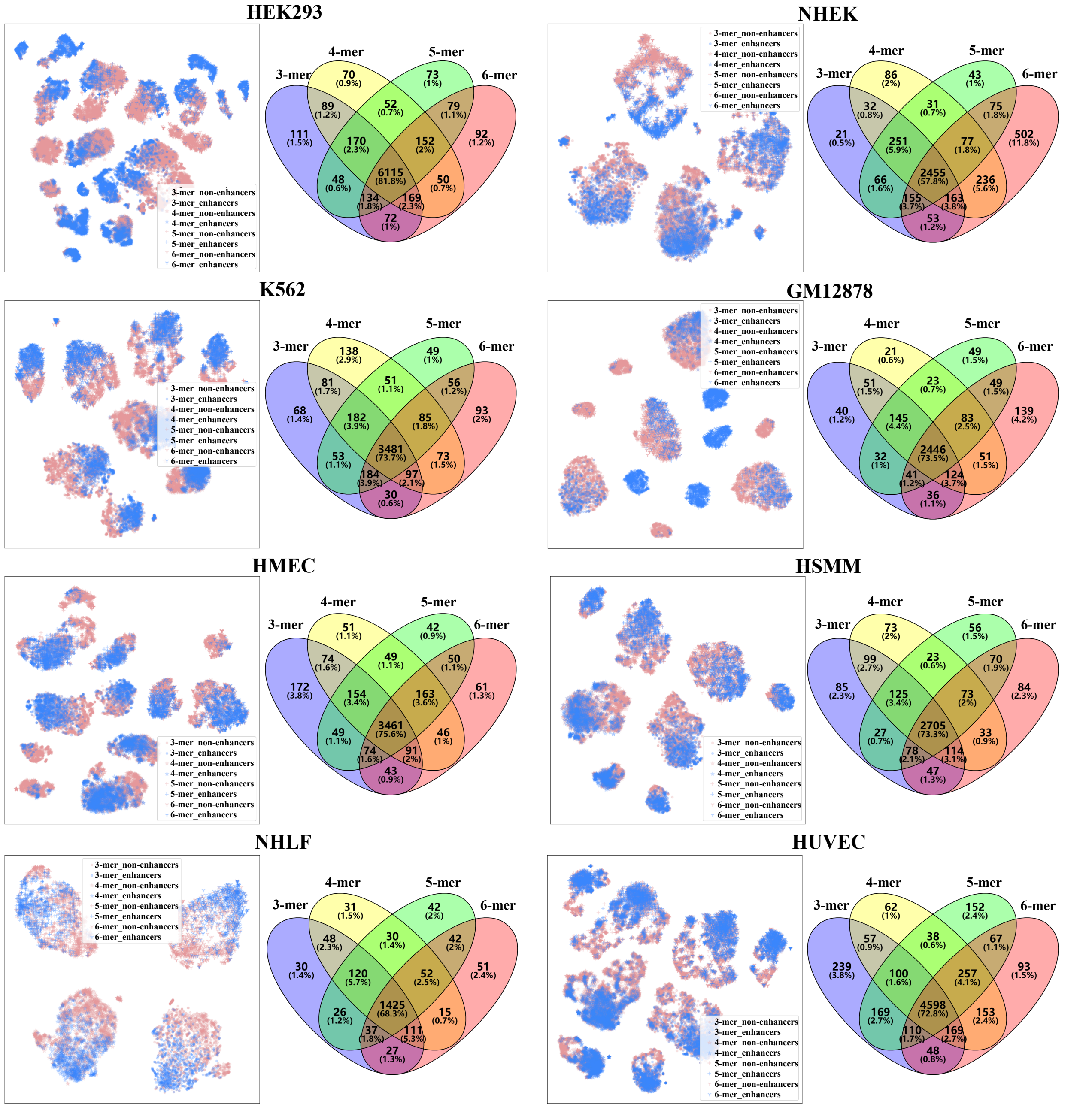

Fig. S2. Complementary analysis of iEnhancer-ELM with multi-scale k-mers (k=3, 4, 5, 6) for eight cell lines in the independent dataset of Basith's dataset. For each cell line, the left subfigure shows the t-SNE visualization of distribution of enhancer and non-enhancer sequences embedded by four iEnhancer-ELM models, and the right subfigure is the Venn diagram of correct prediction by four iEnhancer-ELM models in each cell lines.

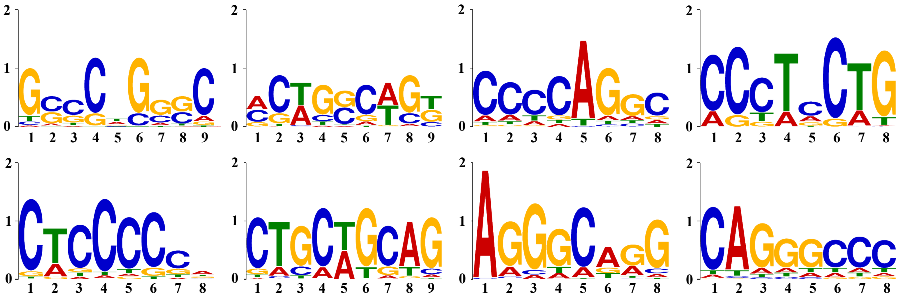

Fig. S3. STREME discovers 8 motifs on the Liu's dataset.

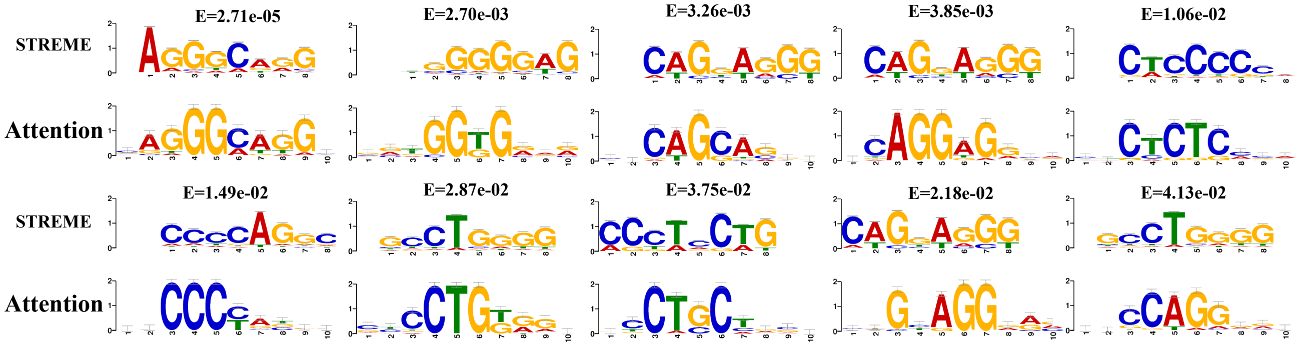

Fig. S4. Comparison between motifs found by attention mechanism and motifs found by STREME. 10 motifs found in our attention mechanism are significantly matched to those found by STREME. And in each subfigure, the top motif (Attention) is found by STREME, and the bottom one is found by our attention mechanism.

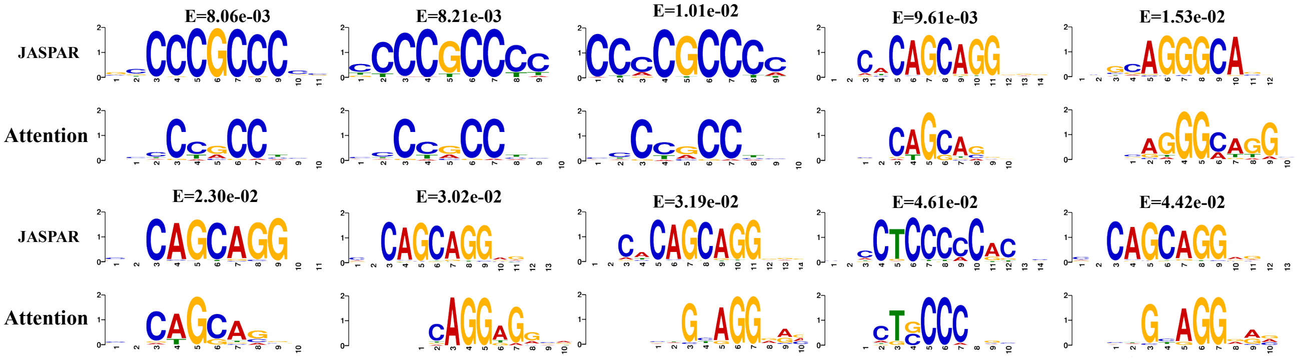

Fig. S5. Comparison between motifs found by attention mechanism and TFBS in JASPAR. In each subfigure, the top motif (Attention) is from JASPAR, and the bottom one is found by attention mechanism.
